# How Determinants of Employee Innovation Behavior Matter During the COVID-19 Pandemic: Investigating Cross-Regional Role *via* Multi-Group Partial Least Squares Structural Equation Modeling Analysis

**DOI:** 10.3389/fpsyg.2022.739898

**Published:** 2022-03-17

**Authors:** Caixia Cao, Michael Yao-Ping Peng, Yan Xu

**Affiliations:** ^1^College of Business, Minnan Science and Technology University, Quanzhou, China; ^2^School of Economics and Management, Foshan University, Foshan, China; ^3^Institute of Educational Administration and Evaluation, University of Taipei, Taipei, Taiwan; ^4^School of Management, Fujian University of Technology, Fuzhou, China

**Keywords:** employee employability, innovation behavior, prior knowledge, perceived organizational support, self-efficacy, job performance

## Abstract

The COVID-19 pandemic cropping up at the end of 2019 started to pose a threat to millions of people’s health and life after a few weeks. Nevertheless, the COVID-19 pandemic gave rise to social and economic problems that have changed the progress steps of individuals and the whole nation. In this study, the work conditions for employees from Taiwan, Malaysia, and the Chinese mainland are explored and compared, and the relationship between support mechanisms and innovation behaviors (IB) is evaluated with a view of the social cognitive career theory. This study adopts the cross-sectional survey and purposive sampling to collect questionnaires. A total of 623 copies of a questionnaire from Taiwanese, 440 copies from Malaysians, and 513 copies from mainlanders were collected in this study to compare the three groups in developing employees’ IBs. Smart-partial least squares for partial least squares structural equation modeling was applied in the structural model to conduct a verification of the hypotheses and comparative analysis in this study. According to the findings, compared with employees from the Chinese mainland, the Taiwanese and Malaysian samples show more significant paths regarding employee employability, IB, prior knowledge, perceived organizational support, self-efficacy, and job performance. Our results will offer more insights and advice concerning theories of human resource.

## Introduction

In the field of human resource administration, the influence of work surroundings and employee employability on innovation behavior (IB) and job performance (JP) has always been a key focus (Amabile and Pillemer, 2012; Chang and Edwards, 2015; Kurtessis et al., 2017; Liu, 2017; Akgunduz et al., 2018). Organizational innovation, or the origin of intrapreneurship dates from employees when they ponder something out of framework at work, propose new ideas, sell or support new individual ideas, and try to seek resources to implement their ideas, that is, to show IBs (Yuan and Woodman, 2010; DE Clercq et al., 2016; Shin et al., 2017). To assist employees in focusing on the process of innovation, one of the major research orientations to organizational creativity is to explore elements that promote and inhibit employees’ IBs (Anderson et al., 2014; Shin et al., 2017). Most of these studies were carried out in complete work surroundings (Lent et al., 2011; Ahmed and Nawaz, 2015; Lamm et al., 2015; Akgunduz et al., 2018; Liguori et al., 2019). Particularly, most of them have verified the importance of online conferences or SNS advisors. Nonetheless, since the COVID-19 pandemic occurring from January 2020 went viral, countries worldwide have begun to stop trading and exchanges, and economic, tourist, and productive exchanges are included, especially work activities. Many economic activities in countries have been ceased and related halting production dates have been extended to inhibit the diffusion of the pandemic. To make it available for employees to go on working while contending with the pandemic, employees began to engage in online work at home, and employees are able to obtain a salary by virtue of technological carriers. However, the influence that employees’ acceptance of working online brings to JP in inadaptable work surroundings remains to be seen (Lamm et al., 2015; Thompson et al., 2017; Akgunduz et al., 2018), especially as each person existing in such an uncertain situation suffers from anxiety and worries (Chang and Edwards, 2015; Schultz et al., 2015; Jemini-Gashi et al., 2019). Thereby, the study aims to explore development of employees’ JP in the case of the global COVID-19 pandemic.

Factors that influence work outcomes from employees (Lent et al., 2011; Ahmed and Nawaz, 2015; Chang and Edwards, 2015; Liguori et al., 2019), or the application effect of work factors (Caesens and Stinglhamber, 2014; Akgunduz et al., 2018) have been explored in most studies on organizational behaviors. Some studies for the past few years began to explore the generation of employees’ IB from the aspect of organizational psychology (Orfila-Sintes and Mattsson, 2009; Amabile and Pillemer, 2012; Liu, 2017). The appearance of positive psychology leads the psychology into a new direction (Lent et al., 2011; Liguori et al., 2019). When employees have a positive perception of the work environment factors of the organization and consider that the organization supports innovation, employees will lean to the direction expected by the organization in terms of motivation and behaviors (Amabile and Pratt, 2016). Based on this view, scholars agree that work environment factors such as organizational encouragement, encouragement from supervisors, support from teamwork, and work autonomy are conducive to creating an atmosphere that supports innovation, thus encouraging members to engage in work and show IB (Kang et al., 2016; Shanker et al., 2017). However, there are still some factors that must exist in the organizational context, but the influence on employees’ IB has not received much attention. Besides, there are diversified ways to comprehend, experience, and seek IB as well in both Western and Asian cultures. For the sake of these, the study aims to explore the enhancement of employees’ IB and the influence on JP.

The social cognitive career theory (SCCT) is conducive to establishing a proper research framework to explore the correlation between work activities, environmental influence factors, and psychological demands (Brown et al., 2011; Chin and Rasdi, 2014; Duffy et al., 2014; Chang and Edwards, 2015; Jemini-Gashi et al., 2019). In conformity with the SCCT, Lent et al. (2002) deemed that a triangular relationship of interaction will be formed by personal attribution, environmental influence factors, and intentional behaviors (Lent et al., 2011; Caesens and Stinglhamber, 2014; Lamm et al., 2015; Jemini-Gashi et al., 2019; Liguori et al., 2019; Meyers et al., 2019). Namely, individual behaviors are produced by the interaction of individuals’ inner minds, feelings, and surroundings (Brown et al., 2011; Chin and Rasdi, 2014; Duffy et al., 2014; Chang and Edwards, 2015). The SCCT architectural pattern shows that an indirect effect of personal cognitive elements occurs between environmental elements and behavioral elements (Lent et al., 2011; Duffy et al., 2014; Thompson et al., 2016; Jemini-Gashi et al., 2019; Liguori et al., 2019). In other words, when personal cognitive elements are expected to influence employees’ IB in a direct way (Ahmed and Nawaz, 2015; Hajizadeh and Zali, 2016; Kurtessis et al., 2017), the effect brought to employees’ IB by externally environmental factors becomes inappreciable (Schultz et al., 2015; Thompson et al., 2016; Liguori et al., 2019; Meyers et al., 2019). Self-efficacy is both the belief of employees in their own successful fulfillment and specific behaviors and competence relevant to the organization (Brown et al., 2011; Chang and Edwards, 2015), and an important element that inspires spontaneous participation and involvement in work (Caesens and Stinglhamber, 2014), and also the core of SCCT (Lent et al., 2011; Thompson et al., 2016; Sheu and Bordon, 2017; Jemini-Gashi et al., 2019; Liguori et al., 2019). Thereby, the combination of cognitive elements and the SCCT between self-efficacy and employees’ IB is suggested to enrich the current literature in the study. Based on the above arguments, this study aims to investigate the relationship between self-efficacy and IB.

Furthermore, in terms of individual cognitive factors, employees will have better performance when they perceive expectation and affirmation from significant others (Lent et al., 2011; Duffy et al., 2014; Hajizadeh and Zali, 2016; Liguori et al., 2019). It has been found by scholars that the interaction that employees have with significant others, such as supervisors and peers, will influence their occupational interests and JP (Brown et al., 2011; Duffy et al., 2014; Ahmed and Nawaz, 2015; Chang and Edwards, 2015; Lamm et al., 2015; Akgunduz et al., 2018). As the profound impact leads to both individual and organizational factors (Cordova et al., 2014; Chang and Edwards, 2015), it has been put forward in this study that prior knowledge (PK) (Ineson et al., 2013; Williams and Lombrozo, 2013; Li et al., 2015; Hajizadeh and Zali, 2016) and perceived organizational support (POS) (Caesens and Stinglhamber, 2014; Ahmed and Nawaz, 2015; Kurtessis et al., 2017; Akgunduz et al., 2018; Meyers et al., 2019) are regarded as crucial individual and organizational cognitive elements to strengthen employees’ skills, and employability is the enhancement of output (Chin and Rasdi, 2014; Cordova et al., 2014; Chang and Edwards, 2015; Akgunduz et al., 2018; Jemini-Gashi et al., 2019; Liguori et al., 2019). It contains the progress of employees for the sake of employment, the increase of their employability, and so on (Ineson et al., 2013; Akgunduz et al., 2018). Regarding the psychological and sociological traits, the study depends on employees’ PK and POS (Ineson et al., 2013; Williams and Lombrozo, 2013; Cordova et al., 2014; Ahmed and Nawaz, 2015; Chang and Edwards, 2015; Li et al., 2015; Jemini-Gashi et al., 2019). Employees’ employability (EE) is affected by employees’ PK and POS, showing that both elements are the most significant resources for employees in terms of further self-efficacy acquisition and EE enhancement. Thus, this study aims to explore the relationships among PK, POS, self-efficacy, and employee employability.

Not only the disparities arising from the pandemic, but also intercultural perspective can be viewed as significant mediating roles that insist on individual feelings and independence (Rehg et al., 2012; Meyers et al., 2019). As cultural boundaries and differences on a global scale become less prominent, the SCCT model that has been put forward to guide such human resource development is more and more applicable. Chinese mainland, Malaysia, and Taiwan were adopted as the research samples for cross-regional comparison to figure out the correlation among the research variables (Hansen et al., 2012; Rehg et al., 2012; Meyers et al., 2019), to explore the disparities of regions in work activities derived from health crisis and cross-culture (Schultz et al., 2015). Some recent studies set about investigating the disparities of countries. For example, Passos et al. (2020) investigated and compared important factors that affect the mental health of the Portuguese and Brazilians; or some scholars only looked into changes to mental conditions, attitudes, and behaviors of employees during the pandemic in a single region (Guzzo et al., 2021; Stergiou and Farmaki, 2021). A comparative study of distinct quarantine policies and pandemic control can offer more diversified insights and understanding for IB of employees. Thus, the study places emphasis on identifying employees’ cognitions of individual and organizational driving elements of EE, self-efficacy, IB, and JP within the organization, and the relationships existing among them (Ahmed and Nawaz, 2015; Akgunduz et al., 2018).

## Literature Review and Hypotheses Development

### Innovation Behavior

According to reviews from Amabile and Pillemer (2012), the main research orientation of early organizational creativity is to discuss creative people’s personality traits or the ability to solve problems with creativity from the aspects of trait or cognitive perspective. Later, social psychologists found from creative people’s autobiographies and letters that creative people are more inclined to produce novel and useful ideas in certain social situations. Thus, scholars’ assessments for individual creativity also gradually shift from the emphasis on individual cognitive competence to the impact of social situational factors on individual creativity performance or IB (Orfila-Sintes and Mattsson, 2009; Chen and Zhou, 2017). Creativity is interpreted from the view of behaviors, which refers to employees who come up with novel or useful ideas, while IB includes the process in which employees propose, introduce, or utilize new ideas in the workplace, and implement creative ideas in different ways with subsequent purposes (Reade and Lee, 2016; Chen and Zhou, 2017). Creativity means that an individual puts forward novel or useful ideas, problem-solving methods, or processes (Amabile, 2011). However, IB refers to employees who propose, introduce, or apply new ideas at work, which will be further implemented or fulfilled through different ways (Yuan and Woodman, 2010; Reade and Lee, 2016). The difference between IB and creativity of employees lies in the fact that IB focuses on the occurrence and implementation of employees’ new ideas. In other words, IB includes creative thinking and concept practice, so creative power or creativity can also be regarded as one of the types of IB (Yuan and Woodman, 2010; Kao et al., 2015). Regarding it from the depth of creativity, it can be divided into big creativity, which changes human life and civilization, and small creativity, which improves the quality of individual work or life and solves daily problems, also known as daily creativity (Conner and Silvia, 2015). Despite members in different professional fields showing unique behaviors of problem discovery and problem solving due to the characteristics of their work situations (Kaufman and Baer, 2005), these behaviors still have common characteristics. For example, employees in daily work ponder something out of framework or reorganize existing ideas, seek or apply new technologies, new procedures, and new approaches at work, figure out creative ideas, sell new ideas to others, and actively strive for resources needed to fulfill new ideas, and plan a timetable to accomplish new ideas (Scott and Bruce, 1994).

For a long time, employees in work surroundings have been struggling with physical and mental stress which keeps employees from coping with learning challenges in a positive manner (Ahmed and Nawaz, 2015). Bewick et al. (2010) argued that British employees were taken as the research object in a study, compared with their peers, they often have considerable pressure on loans, life, and performance, and it was emphasized that scholars are necessarily supposed to shift the focus from work performance to the exploration of employees’ psychological issues (Kurtessis et al., 2017; Meyers et al., 2019). Even though scholars have explored employees’ IB from different levels, some research gaps still exist which are worthy of being explored and discussed, such as how IB develop, and internal and external elements influencing employees’ IB (Reade and Lee, 2016; Chen and Zhou, 2017; Baradarani and Kilic, 2018). Besides, Folkman and Moskowitz (2000) indicated in their study that the subsequent research needs to emphasize the discussion of positive emotions and IB (Kurtessis et al., 2017) as figuring out relevant elements available to keep down psychological health problems arising from stress in as an effective way as possible, if it is not explored from the aspect of positive results (Thompson et al., 2016). Thus, based on the SCCT, the study adopts IB as the outcome variable for the exploration of the effect that correlative factors bring to it. Figuring out different mechanisms conducive to employees’ IB is in the interest of organizational behaviors and administrators.

*H1: IB plays a positive and significant effect on employees’ JP.*

### Employee Employability

For the past few years, scholars have been more committed to conducting research related to employability (Ineson et al., 2013; Thompson et al., 2016). The concepts and operations of industrial organizations worldwide have been modified by the substantial technological, social, and economic vicissitudes that have sprung up in recent decades (Abbas et al., 2015; Akgunduz et al., 2018; Abbas and Sağsan, 2019). Therefore, the highest standards of human capital development are guaranteed by dynamic organizations, which make contributions to economic progress (Ahmed et al., 2015; Baek and Cho, 2018). Scholars have conducted a study on the implications of EE and the causal relationships between EE and other factors (Hennemann and Liefner, 2010; Thompson et al., 2016; Baek and Cho, 2018) by the ways of research situations and method design, as well as the integration of theoretical and practical analysis (Ineson et al., 2013). Van Der Heijde and Van Der Heijden (2006) stated that EE is the proper application of individual capabilities (Pan and Lee, 2011; Blázquez et al., 2018), constant acquisition and creation of necessary occupational skills to fulfill all the tasks, and adapt to internal and external changes in the job market (Chang and Edwards, 2015; Akgunduz et al., 2018). Thereby, the demand for a critical and reflective mind, capabilities of solving problems, self-government, learning, and related capabilities are constantly enhanced in an interdisciplinary way (Ineson et al., 2013; Thompson et al., 2016; Makkonen and Olkkonen, 2017). Some previous studies have stated that, besides the influence brought to EE by basic education, elements such as individual conditions, interpersonal relationships, and external elements that are not accessible in human resources need to be taken into consideration as well. Pan and Lee (2011) conducted a survey of the samples in Taiwan and adopted the scale of employment from Andrews and Higson (2008), who suggested that employability necessarily involves the general and professional capabilities required at work, attitude to work, occupational plan capabilities, and confidence. The classification of employability made by Pan and Lee (2011) is taken as the measure for EE in this study.

According to De Cuyper et al. (2008), EE is of great importance in the society of post-industrial knowledge, which constantly updates knowledge to keep competitive in a worldwide market and makes them accessible to handling temporary and subsequent development—new psychological contracts developed by individuals tend to enhance their IB (Lent et al., 2011; Ahmed and Nawaz, 2015; Akgunduz et al., 2018). Besides, with less time, related experience, skills, and knowledge that have been updated, individuals are available to process the same things and tasks in a more effective way (Lent et al., 2011; Chang and Edwards, 2015) —and a social network that has undergone positive development—to increase EE. Abundant time saved will be contributed to life needs and individual planning for future, thus strengthening IB (Thompson et al., 2016). Likewise, higher employability can enable employees to contend with job challenges in the future with a broader view. They not only master the content of organizational tasks, but also show a more precise direction for planning and preparing for tasks to be accomplished (Ineson et al., 2013; Chang and Edwards, 2015), thus keeping down their insecurity and improving IB. Based on the above phenomena, the study proposes the following hypothesis:

*H2: EE plays a positive and significant effect on employees’ IB.*

### Self-Efficacy

According to SCCT scholars, both environmental factors and cognitive factors in a certain context, particularly those beliefs leading to success and behavior, will influence individuals’ behavioral outcomes (Brown et al., 2011; Chin and Rasdi, 2014; Chang and Edwards, 2015; Liguori et al., 2019). These beliefs are called “self-efficacy” by them, namely a significant cognitive variable in individual factors during accounting for individual behaviors (Caesens and Stinglhamber, 2014), and interaction with the surroundings (Lent et al., 2011; Duffy et al., 2014; Chang and Edwards, 2015; Jemini-Gashi et al., 2019). It can also be regarded as the foundation for the motivation of human behaviors (Cordova et al., 2014), mental health, and individual accomplishments (Lent et al., 2011; Liguori et al., 2019). The field of human resources takes a wide application of self-efficacy to probe into the psychological cognitive factors of employees in different situations and their positive impact on task accomplishment and employees’ occupational development (Brown et al., 2011; Caesens and Stinglhamber, 2014; Duffy et al., 2014; Jemini-Gashi et al., 2019). To have a clearer understanding of the application of SCCT, major findings of relevant studies are described and summarized as follows in Table 1.

**TABLE 1:** Summary of related literatures.

Authors	Variables	Location	Findings
Duffy et al., 2014	Work volition, self-efficacy, outcome expectations, interests, goals	United States	Work volition was a significant moderator in the link of self-efficacy and outcomes expectations and self-efficacy and goals
Chang and Edwards, 2015	Job satisfaction, self-efficacy, coping	Taiwan	Self-efficacy was positively associated with problem-focused coping style and job satisfaction and negatively associated with emotion-focused coping
Lent et al., 2016	Self-efficacy, outcome expectations, social support, conscientiousness, exploration goals, prior engagement, anxiety	United States	Self-efficacy related strongly to outcome expectations, social support, conscientiousness, exploration goals, prior engagement in career exploration, decisional anxiety, and level of career decidedness
Jemini-Gashi et al., 2019	Career self-efficacy, social support, career indecision	Kosovo	Social support was indirectly correlated with career indecision, as career self-efficacy played a mediating role in this relationship
Liguori et al., 2019	Entrepreneurial intentions, entrepreneurial attitudes, entrepreneurial outcome, expectations	United States	The significant role of entrepreneurial attitude in mediating the relationship between entrepreneurial motivation and intention
Li et al., 2019	Protean career orientation, career decidedness, career decision self-efficacy, career adaptability	Hong Kong and the United States	Protean career orientation is positively related to career decision self-efficacy and career adaptability

Based on the above discussion, it is considered that employees having confidence in their capabilities will lead to behaviors that are more efficient and interpersonal relationships that are better than whose who lack confidence (Brown et al., 2011; Chin and Rasdi, 2014; Chang and Edwards, 2015). As Chin and Rasdi (2014) considered, employees who are highly self-motivated seek resources and opportunities to fulfill tasks existing in a social network (Lent et al., 2011; Thompson et al., 2016). Only by establishing and insisting on network relationships can they achieve their goals. Knowledge and resources are in need (Lent et al., 2011; Jemini-Gashi et al., 2019). Moreover, teamwork can also be viewed as a strong network relationship, and the process in which problem solving and task fulfillment are conducted for employees via teamwork will play a positive effect on their EE (Duffy et al., 2014; Chang and Edwards, 2015). Given the above, this study puts forward H2 as follows:

*H3: Self-efficacy plays a positive and significant effect on EE.*

Some scholars have attached their research to the concerns for psychological health, POS (Chin and Rasdi, 2014), and lifestyles for employees (Lent et al., 2011). Nonetheless, few studies yet have addressed general self-efficacy and IB in this population. Research results from Jemini-Gashi et al. (2019) showed that individuals express a lower support level, limited sources that support comes from, and low perceived support (Brown et al., 2011). According to Caesens and Stinglhamber (2014), employees who have a high level of self-efficacy are inclined to gain diversified benefits at work which eventually give rise to a higher level of work satisfaction. It indicates that employees’ failure to receive timely and necessary mental support when encountering work pressure leads to the deduction in employees’ general self-efficacy and IB (Thompson et al., 2016). Besides, it might be conducive to unique stressors. On the contrary, employees owning higher self-efficacy show a higher level of IB. In a word, the study deduces H4:

*H4: Self-efficacy plays a positive and significant effect on employees’ IB.*

### Developing Innovation Behavior in Human Resources

Two causal mechanisms are conducive to the development of IB in human resources, and they are PK, as well as POS. With support for IB establishment, organizations or supervisors have access to devising the organizational context, including individual and organizational factors (Chin and Rasdi, 2014; Chang and Edwards, 2015; Thompson et al., 2016; Liguori et al., 2019) to improve the efficiency and responsiveness of knowledge acquisition. According to scholars, organizations or supervisors claimed to make use of, integrate, and rearrange individual and organizational elements to establish an optimal organizational environment for building employees’ IB (Lent et al., 2011; Chin and Rasdi, 2014; Ahmed and Nawaz, 2015; Kurtessis et al., 2017; Akgunduz et al., 2018). Organizations or supervisors carry out a range of support activities to identify individual and organizational elements (Thompson et al., 2016; Liguori et al., 2019), where PK focuses on perceiving knowledge and skills that internally exist (Ineson et al., 2013; Williams and Lombrozo, 2013; Cordova et al., 2014; Li et al., 2015; Hajizadeh and Zali, 2016) and POS focuses on offering tangible and intangible resources to accelerate employees’ capabilities to fulfill their tasks or goals (Caesens and Stinglhamber, 2014; Ahmed and Nawaz, 2015; Lamm et al., 2015; Liguori et al., 2019). In this study, a better way to construct IB to make support activities adapted to PK and POS is considered.

#### Building a Support Mechanism for Innovation Behavior: Prior Knowledge

People’s interpretation of existing situations and information relies on self-perception. Based on self-perception, people are accessible to identifying things and the environment in which they are living (Chang and Edwards, 2015). Self-perception assists learners while they are learning, but the learners may not realize it (Ineson et al., 2013; Thompson et al., 2016). The prior capability makes the learner available to comprehend external knowledge and information and then integrate the knowledge connotation obtained with the learner’s prior capability (Williams and Lombrozo, 2013; Li et al., 2015), thus producing more abundant basis of prior capabilities (Ineson et al., 2013; Cordova et al., 2014). Thus, the prior capability is not immutable, but can enhance over time, revealing path-dependent characteristics (Williams and Lombrozo, 2013; Li et al., 2015), and the PK can be enhanced with the attitude to learning and the learner’s motivation (Ineson et al., 2013; Cordova et al., 2014; Hajizadeh and Zali, 2016; Liguori et al., 2019).

Based on various theories, the effect of PK has been explored by scholars in studies on PK (Cordova et al., 2014; Hajizadeh and Zali, 2016). Despite some empirical studies that stated that the effect brought to employee performance by PK does not exist, some scholars still consider that PK is significantly correlated with learning (Cordova et al., 2014). Referring to the theory of cognitive load, Amadieu et al. (2009) have explored the effect of staff’s PK in acquiring electronic documents that are internal within the organization (Williams and Lombrozo, 2013). It is concluded that a high degree of PK can make the staff more capable of information processing and learning route arrangement with their own mental model (Williams and Lombrozo, 2013; Hajizadeh and Zali, 2016; Liguori et al., 2019). In addition, a high degree of PK can make the staff unlikely to contend with work confusion than those who have a low degree of PK (Ineson et al., 2013; Liguori et al., 2019). It is possibly attributed to the fact that the explicit and written knowledge is of limited use, even though the staff has a high degree of PK of this kind (Williams and Lombrozo, 2013; Hajizadeh and Zali, 2016). However, the implicit and complicated knowledge will facilitate the employees who have a high degree of PK as such kind to probe into the knowledge connotation in a more careful and deep way (Cordova et al., 2014), which contributes to shifting this process of exploration into their own EE. In a word, the study puts forward hypotheses as follows:

*H5: PK plays a positive and significant effect on EE.*

Employees who have more PK drive themselves to gain more external knowledge to figure out work problems and challenges, thus fulfilling individual goals and strengthening the individual perception of accomplishment (Williams and Lombrozo, 2013; Cordova et al., 2014). In other words, employees who strengthen their own capabilities by learning, perceiving, and combining diversified knowledge possess more PK during fulfilling tasks (Ineson et al., 2013; Hajizadeh and Zali, 2016). This contributes to enhancing individual feelings of IB. Employees who own more PK will have access to the identification of valuable and helpful information and knowledge to handle more business in the external environment, thus playing an effect on work satisfaction and efficiency. In some past studies, it is stated that expecting there to be significant and immediate self-efficacy change makes sense (Ineson et al., 2013; Liguori et al., 2019), accompanied by vital improvement of PK as time passes for employees (Cordova et al., 2014). Similarly, faced with negative environmental events or the need of assistance, employees can alleviate the influence arising from negative environmental events by means of the accumulated knowledge or resources (Hajizadeh and Zali, 2016). When intense pressure comes to employees, and they feel vital resources are lost, an effect brought to employees’ estimation of stress situations will occur if they have adequate PK, thus resulting in the reduction of adaptive strategies for negative feelings and inappropriate utility (Ineson et al., 2013). Thus, H6 is proposed in this study as follows:

*H6: PK plays a positive and significant effect on employees’ self-efficacy.*

#### Building Support Mechanism for Innovation Behavior: Perceived Organizational Support

Perceived organizational support (Ahmed and Nawaz, 2015; Akgunduz et al., 2018) refers to how employees perceive whether an organization is concerned with their IB and dedications (Gillet et al., 2012; Caesens and Stinglhamber, 2014; Demir, 2015) or whether the organization assists them in fulfilling professional and individual goals (Uppal and Mishra, 2014; Kurtessis et al., 2017; Liguori et al., 2019). When positive organizational support comes to employees, more job security and involvement in work come to them (Kose, 2016; Kurtessis et al., 2017). POS has a strong correlation with many positive traits and behaviors in the workplace, and a positive organizational atmosphere (Ahmed and Nawaz, 2015; Kose, 2016; Jemini-Gashi et al., 2019) and a positive organizational citizenship behavior are included (Caesens and Stinglhamber, 2014; Demir, 2015; Lamm et al., 2015; Akgunduz et al., 2018). These associations mostly seem to be correlated with other variables in this study (Meyers et al., 2019). For example, according to Kose (2016), organizational citizenship behavior is regarded as an intention for employees to assist others beyond the range of their assigned responsibilities, and it seems to be approximated to a social dimension of self-efficacy and EE.

There is a crucial relationship between POS and self-efficacy which has been discussed in past studies (Caesens and Stinglhamber, 2014; Kose, 2016). When employees feel as if the organization cares about their well-being, they provide their contributions in exchange. POS also enhances employees’ sense of belonging (Demir, 2015; Lamm et al., 2015; Akgunduz et al., 2018). Regarding the relationship between POS and self-efficacy, Kose (2016) stated that employees with the perception of organizational support frequently feel security in their positions and consider that their organizations care for their professional advancement (Lent et al., 2011; Uppal and Mishra, 2014; Schultz et al., 2015; Kurtessis et al., 2017). It is reasonable that employees considering their organizations are concerned about their individual and professional life would have an intention of searching for more resources to accomplish tasks or obtain more duties (Akgunduz et al., 2018), which are shown as dimensions of self-efficacy and EE (Lent et al., 2011; Caesens and Stinglhamber, 2014). POS is positively correlated with organizational citizenship behaviors (Demir, 2015; Meyers et al., 2019), and it offers a prediction of more helping behaviors within an organization.

For employees, POS is viewed as the most direct and efficient support source (Akgunduz et al., 2018). Organizations offer assistance to employees when it comes to job demands and problem solving, as well as anxiety led by the utilization of technological tools at work (Lent et al., 2011; Lamm et al., 2015). In addition, by means of the support for effective work from the organization, the state of job engagement will be enhanced, and the employees’ successful accomplishment of tasks will be improved (Kurtessis et al., 2017; Jemini-Gashi et al., 2019; Liguori et al., 2019). Akgunduz et al. (2018) stated that employees with adequate competence and motivation are able to fulfill their organizations’ targets and perform as required when no manager supervises (Meyers et al., 2019). POS shows a relationship with theories of social interaction (Ahmed and Nawaz, 2015; Kurtessis et al., 2017). Favorable work environments, which are integrated with psychological characteristics of employees, can be generated to provide employees with more confidence in job task accomplishment (Caesens and Stinglhamber, 2014; Liguori et al., 2019). Employees will have more motivation to participate in work targets and get to know values and insights derived from task fulfillment and problem solving (Lent et al., 2011; Ahmed and Nawaz, 2015), thus enhancing employee self-efficacy, if they feel that the POS from supervisors and peers has built the positive psychological surroundings. Thus, H7 is proposed in this study as follows:

*H7: POS has a positive and significant effect on employees’ self-efficacy.*

Furthermore, the POS, accompanied by its relationship with EE, contributes to accelerating the work interest for employees and the utilization of their occupational skills (Caesens and Stinglhamber, 2014; Ahmed and Nawaz, 2015), and further strengthening employees’ capabilities (Lent et al., 2011; Liguori et al., 2019). When employees encounter practical problems, like critical analysis, problem resolution, and reflection, they can exhibit better attitudes to work and capabilities of a critical mind (Schultz et al., 2015; Jemini-Gashi et al., 2019). Akgunduz et al. (2018) claimed that employees obtain support that supervisors or organizations offer, and the support can accelerate the creativity for employees, thus accelerating their employment skills (Gillet et al., 2012; Caesens and Stinglhamber, 2014). Mulholland and O’Connor (2016) presented a confirmation that employees taking in the POS pattern will alter their occupational skills, attitudes, and behaviors to strengthen their critical mind, autonomy, and capabilities related to employment. Thus, H8 is proposed in this study as follows:

*H8: POS plays a positive and significant effect on EE.*

**FIGURE 1:**
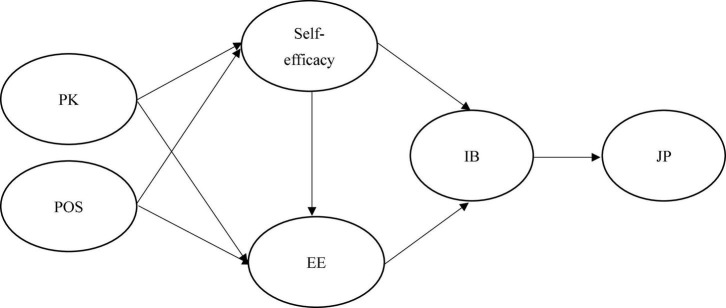
Research framework. PK, prior knowledge; POS, perceived organizational support; EE, employee employability; IB, innovation behavior; JP, job performance.

Given the above hypotheses, this study puts forward the research framework in Figure 1 as follows.

## Methodology

### Sampling

The purpose of this research is to explore the employee IB, and to analyze the impact of internal and external factors provided by the organizations and individual knowledge base. The research sample in this study comprised employees. Purposive sampling was adopted. However, this sampling suffers from several disadvantages. Vulnerability to errors in judgment by researchers, low level of reliability and high level of bias, and inability to generalize research findings are three main disadvantages. To avoid these disadvantages, some conditions were set during sampling in this study to make the samples obtained better conform to sample reliability and to improve the generalization of the study. The results indicated that subject did not significantly impact the research variables, and so did not need to be included as an independent variable in subsequent analyses. To discuss IB of employees in a more clarified manner, not all the employees are regarded as the study population, but only the employees in the information service industry. Moreover, while filling the questionnaire, all the samples were already at work, rather than being isolated at home. In the questionnaire, participants were informed of the research purpose, research ethics, and low risks, and the questionnaire information was processed in an anonymous way. The response period ran from May 2020 to August 2020. Since different pandemic prevention policies were adopted in Taiwan, mainland China, and Malaysia, we try to explore whether these pandemic prevention policies have had a different impact on attitudes and behaviors of employees during this period. This study constructed a structural model to explore the correlations among POS, PK, self-efficacy, EE, IB, and JP. It sampled from Taiwanese, Malaysian, and mainland China companies. This study selected more than 20 Taiwanese, Malaysian, and mainland China companies, and then sent 1000 questionnaires to each of them. Finally, a total of 640 Taiwanese questionnaires, 450 Malaysian questionnaires, and 568 mainland China questionnaires were returned, for an effective response rate of 64.0, 45.0, and 56.8%. In the Taiwanese sample, most are men (61.8%), whose level of education is mostly undergraduate or above (78.9%), and most of them are between 30 and 40 years old (77.3%) with an average working year of 3.9. In the Malaysian sample, most are men (55.4%), whose level of education is mostly undergraduate or above (68.4%), and most of them are between 35 and 40 years old (43.2%) with an average working year of 5.2. In the sample of mainland China, most are men (62.1%), whose level of education is mostly undergraduate or above (66.9%), and most of them are between 30 and 35 years old (53.8%) with an average working year of 4.3.

This study hid the names of constructs and assigned the question items randomly to prevent common method variance (CMV). The Harman one-factor analysis method as used to test for CMV. The explained variance in one factor was 35.27%, which is smaller than the recommended threshold of 50%. Therefore, CMV was not problematic in this study (Podsakoff and Organ, 1986). Before conducting hypotheses testing, this study must ensure that the values of the variance inflation factor (VIF) are less than 5, but the research results showed that the VIF values were between 1.332 and 2.723. Thus, there were no multicollinearity problems among the latent variables (Hair et al., 2017).

### Measures

Most of the scales in the questionnaire are adopting previous studies and modified to suit the research context. In PK, 10 items were developed based on a prior scale proposed by Silva et al. (2013). To divide POS into supervisor and colleague support (four items) and organizational support (eight items), we adopted the scale proposed by De Vos et al. (2011). In employee employability, the scales proposed by Pan and Lee (2011) were adopted, including general ability for work (GAW) (eight items), professional ability for work (PAW) (four items), attitude at work (AW) (three items), and career planning and confidence (CPC) (three items). For self-efficacy, the scale is revised and integrated with six items developed by Rigotti et al. (2008). IB was measured using Kao et al.’s (2015) instrument, which comprehensively assesses IB in three items. For JP, five items were selected based on Janssen’s (2001) scale. All items were measured with a 5-point Likert scale (1 = totally disagree; 5 = totally agree) and are shown in Table 2.

**TABLE 2:** Instruments description.

Construct	Variables	Items
Prior knowledge	Prior knowledge	Enough knowledge to solve problem
		Enough knowledge to plan
		Self-awareness
		Enough knowledge to make critical analysis
		Enough knowledge to make decision
		Self-management
		Global awareness
		Enough knowledge to apply subject understanding
		Teamwork
		Willingness to learn
Perceived organizational support	Supervisor and colleague support	My boss regularly gives me feedback about my performance.
		My boss makes sure that I can learn on the job by giving me challenging assignments.
		My colleagues regularly give me feedback about my performance.
		My boss makes sure that I develop the competencies that I need for my career.
	Organizational support	I get the necessary time and means to further develop my competencies.
		I can make use of a personal development plan to know what competencies I need to develop and how I can develop them best.
		My organization provides new and creative training opportunities.
		I can regularly change jobs within my company (without promotion) to develop new competencies.
		All information about career opportunities in the organization is readily available.
		I have been given tasks that develop my competencies for the future.
		I have been given a personal development plan to better understand my possibilities within the organization and the competencies I need to fully exploit them.
		I have been given the possibility within my organization to develop the competencies I need to get a promotion and move to a function at a higher level of the organization.
Self-efficacy	Self-efficacy	I can remain calm when facing difficulties in my job because I can rely on my abilities.
		When I am confronted with a problem in my learning tasks, I can usually find several solutions.
		Whatever comes my way in my learning tasks, I can usually handle it.
		My past experiences in my learning tasks have prepared me well for my occupational future.
		I meet the goals that I set for myself in my learning tasks.
		I feel prepared for most of the demands in my learning tasks.
Employability	General ability for work	Expression and communication.
		Time management.
		Leadership.
		Innovation.
		Teamwork.
		Native language.
		Foreign language.
		Stability and pressure resistance.
	Professional ability for work	Professional knowledge and skill.
		Computer literacy.
		Application of theory to work.
		Problem finding and solving.
	Attitude at work	Learning desire.
		Plasticity.
		Understanding of professional ethics.
	Career planning and confidence	Understanding and planning of individual career development.
		Understanding of environment and development of industries.
		Job search and self-promotion.
Innovation behavior	Innovation behavior	I often come up with new and practical ideas to improve performance.
		I often develop new methods for work implementation.
		I often use new technologies, processes, and techniques in information service.
Job performance	Job performance	I always complete the duties specified in my job description.
		I fulfill all responsibilities required by my job.
		I never fail to perform essential duties.
		I never neglect aspects of the job that I am obligated to perform.
		I meet all the formal performance requirements of the job.

## Results

### Evaluation of the Measurement Model

All scales used in this study were found to be reliable, with Cronbach’s α ranging from 0.83 to 0.96. Table 3 shows the reliability of each scale, and the factor loadings for each item therein. To gauge validity, this study employed confirmatory factor analysis (CFA) using AMOS 23.0 to verify the construct validity (both convergent and discriminant) of the scales. According to Hair et al.’s (2010) recommended validity criteria, CFA results show standardized factor loading of higher than 0.5; average variance extracted (AVE) ranges between 0.514 and 0.803; and composite reliability (CR) ranges between 0.863 and 0.962. All three criteria for convergent validity were met, and correlation coefficients were all less than the square root of the AVE within one dimension, suggesting that each dimension in this study had good discriminant validity.

**TABLE 3:** Measurement properties.

	1	2	3	4	5	6	7	8	9	12
(1) PK	*0.817*									
(2) Organization	0.525	*0.837*								
(3) Supervisor	0.501	0.866	*0.877*							
(4) Self-efficacy	0.405	0.537	0.508	*0.779*						
(5) GAW	0.389	0.345	0.333	0.391	*0.718*					
(6) PAW	0.529	0.347	0.322	0.389	0.538	*0.832*				
(7) AW	0.599	0.439	0.414	0.442	0.525	0.767	*0.834*			
(8) CPC	0.681	0.518	0.487	0.430	0.450	0.614	0.713	*0.895*		
(9) IB	0.446	0.658	0.603	0.521	0.308	0.331	0.417	0.434	*0.896*	
(10) JP	0.512	0.736	0.665	0.526	0.330	0.353	0.419	0.471	0.643	*0.789*
Mean	3.572	3.750	3.658	3.793	3.568	3.691	3.685	3.624	3.933	3.833
*SD*	0.692	0.729	0.754	0.544	0.606	0.671	0.673	0.731	0.714	0.659
α	0.944	0.939	0.900	0.872	0.817	0.852	0.780	0.876	0.923	0.836
AVE	0.668	0.701	0.770	0.607	0.515	0.692	0.695	0.801	0.803	0.623
CR	0.952	0.949	0.962	0.902	0.863	0.900	0.872	0.923	0.913	0.889

*The italized values mean squared root of AVE values.*

### Inner Model Analysis

Before proceeding to examine the structural model, we first tested model fit. Henseler et al. (2015) proposed three model fitting parameters: the standardized root mean square residual (SRMR), the normed fit index (NFI), and the exact model fit. In this study, the SRMR value was 0.054 (<0.08) and the NFI was 0.932 (>0.90) and the *d*_*ULS*_ < bootstrapped HI 95% of *d*_*ULS*_ and *d*_*G*_ < bootstrapped HI 95% of *d*_*G*_ indicating the data fits the model well. Partial least squares structural equation modeling (PLS-SEM) was adopted to construct the structural model; specifically, verification of the structural model was performed using SmartPLS 3.0 (path analysis). To assess the structural model, Hair et al. (2017) suggested looking at the *R*^2^, beta (β), and the corresponding *t*-values via a bootstrapping procedure with a resample of 5000. They also suggested that in addition to these basic measures, researchers should also report the predictive relevance (*Q*^2^) as well as the effect sizes (*f*^2^). Prior to hypotheses testing, the values of the variance inflation factor (VIF) were determined. The VIF values were less than 5, ranging from 1 to 1.914. Thus, there were no multicollinearity problems among the predictor latent variables (Hair et al., 2017).

**FIGURE 2:**
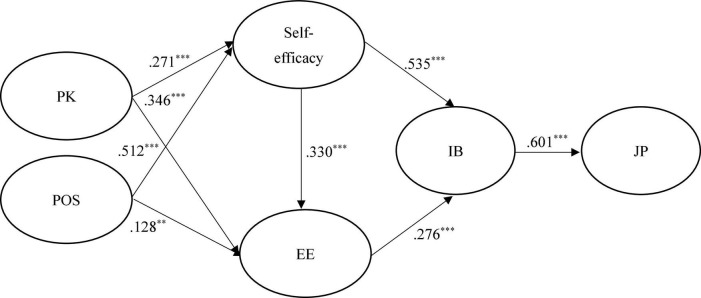
Structural model on Taiwanese employees. PK, prior knowledge; POS, perceived organizational support; EE, employee employability; IB, innovation behavior; JP, job performance. ***p < 0.001.

**FIGURE 3:**
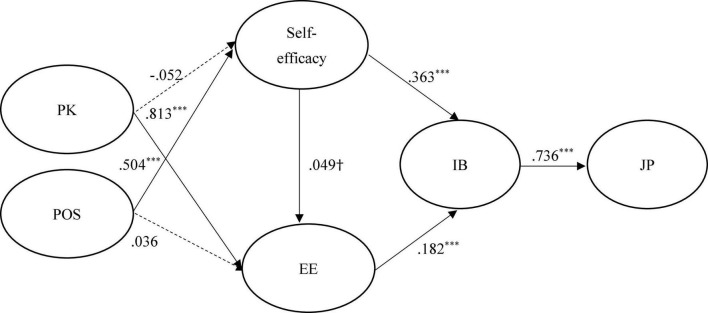
Structural model on mainland China employees. PK, prior knowledge; POS, perceived organizational support; EE, employee employability; IB, innovation behavior; JP, job performance. ***p < 0.001 and ^†^*p* < 0.1.

**FIGURE 4:**
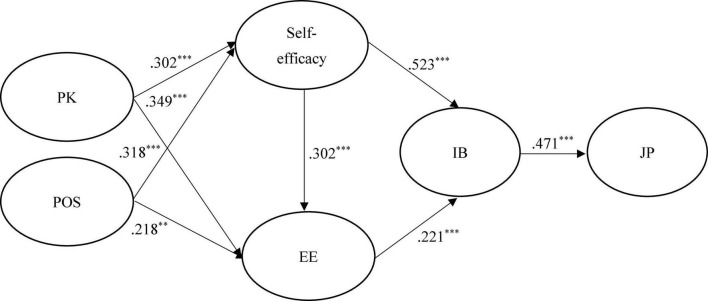
Structural model on Malaysian employees. PK, prior knowledge; POS, perceived organizational support; EE, employee employability; IB, innovation behavior; JP, job performance. ***p < 0.001.

Figures 2–4 show the results of the hypothesized relationships and standardized coefficients in Taiwanese and mainland China samples. The results showed that IB was positively and significantly related to JP (β_Taiwan_ = 0.601, *f*^2^ = 0.566, *p* < 0.001; β_China_ = 0.736, *f*^2^ = 1.185, *p* < 0.001; β_Malaysia_ = 0.471, *f*^2^ = 0.285, *p* < 0.001), supporting H1; respectively, we have found comparable results with Baradarani and Kilic (2018). Self-efficacy (β_Taiwan_ = 0.535, *f*^2^ = 0.399, *p* < 0.001; β_China_ = 0.363, *f*^2^ = 0.159, *p* < 0.001; β_Malaysia_ = 0.523, *f*^2^ = 0.353, *p* < 0.001) and EE (β_Taiwan_ = 0.276, *f*^2^ = 0.106, *p* < 0.001; β_China_ = 0.182, *f*^2^ = 0.040, *p* < 0.001; β_Malaysia_ = 0.221, *f*^2^ = 0.063, *p* < 0.001) were also positively and significantly related to IB, supporting H2 and H4. The findings of this research confirm consistent with findings of Wei et al. (2020) that self-efficacy plays a significant role in the development of IB.

In addition, self-efficacy (β_Taiwan_ = 0.330, *f*^2^ = 0.106, *p* < 0.001; β_China_ = 0.049, *f*^2^ = 0.040, *p* < 0.1; β_Malaysia_ = 0.302, *f*^2^ = 0.063, *p* < 0.001) was positively and significantly related to EE in Taiwanese and Malaysian samples rather than in the mainland China sample, partial supporting H3. This is consistent with the results of Liu et al. (2020) and Zhao et al. (2021) that found a positive effect of self-efficacy on employability. Similarly, the paths of PK → self-efficacy (β_Taiwan_ = 0.271, *f*^2^ = 0.104, *p* < 0.1; β_China_ = –0.052, *f*^2^ = 0.003, *p* > 0.1; β_Malaysia_ = 0.302, *f*^2^ = 0.110, *p* < 0.001) and POS → EE (β_Taiwan_ = 0.128, *f*^2^ = 0.016, *p* < 0.1; β_China_ = 0.036, *f*^2^ = 0.003, *p* > 0.1; β_Malaysia_ = 0.218, *f*^2^ = 0.069, *p* < 0.001), showed that the relations were positive and significant in Taiwanese and Malaysian samples rather than in the mainland China sample, therefore, partially supporting H6 and H8. A similar result was found in a study of United Kingdom, Australia, and Switzerland institutions, where PK was found to have a positive influence on self-efficacy (Ineson et al., 2013).

Finally, the paths of PK → EE (β_Taiwan_ = 0.271, *f*^2^ = 0.150, *p* < 0.1; β_China_ = –0.052, *f*^2^ = 0.945, *p* > 0.1; β_Malaysia_ = 0.302, *f*^2^ = 0.179, *p* < 0.001) and POS → self-efficacy (β_Taiwan_ = 0.512, *f*^2^ = 0.370, *p* < 0.1; β_China_ = 0.504, *f*^2^ = 0.306, *p* > 0.1; β_Malaysia_ = 0.318, *f*^2^ = 0.122, *p* < 0.001) showed that the relations were positive and significant in both samples, supporting H5 and H7. The Stone–Geisser Q2 values obtained through the blindfolding procedures for self-efficacy (*Q*^2^ = 0.178), EE (*Q*^2^ = 0.335), IB (*Q*^2^ = 0.352), and JP (*Q*^2^ = 0.303) were larger than zero, supporting the model has predictive relevance (Hair et al., 2017).

### Multiple Group Analysis: Taiwan, Mainland China, and Malaysia

It was confirmed that the measurement pattern was stable. However, to avoid overgeneralizing the data-driven patterns and theories, the study followed the suggestion of Hair et al. (2010) to divide the sample data into three groups based on regions (623 Taiwanese, 440 Malaysian, and 513 mainland China employees, respectively). The partial measurement invariance was established that was the basic requirement to compare as well as interpret the PLS-SEM’s findings for examining the specific Multiple Group Analysis (MGA) group’s differences (Henseler et al., 2016). Table 4 indicates the structural models’ results and MGA by using non-parametric methods including Henseler’s MGA as recommended by Henseler et al. (2009). Despite several differences in terms of significant path estimates between the groups, as indicated in Table 4, the multi-group permutation tests (right column) showed there are seven significant differences between the two groups on all paths. The results signify that the region plays a moderating role on the relationship among PK, POS, self-efficacy, SE, IB, and JP (Hair et al., 2017). The differences in paths comparison among Taiwan vs. mainland China, Taiwan vs. Malaysia, and Malaysia vs. mainland China show that four paths, two paths, and five paths were significant sequentially. These results imply that the research framework did differ among the three regions.

**TABLE 4:** Multi-group analysis result.

Paths	—β_Taiwan_ -β_China_—	*p*-value Henseler’s MGA	—β_Taiwan_ -β_Malaysia_—	*p*-value Henseler’s MGA	—β_Malaysia_ -β_China_—	*p*-value Henseler’s MGA
H1: IB → JP	0.135	0.000	0.129	0.019	0.264	0.000
H2: Self-efficacy → IB	0.172	0.999	0.012	0.421	0.160	0.996
H3: Self-efficacy → EE	0.281	0.000	0.027	0.328	0.254	0.000
H4: EE → IB	0.095	0.750	0.055	0.183	0.040	0.750
H5: PK → EE	0.468	0.000	0.003	0.513	0.465	0.000
H6: PK → Self-efficacy	0.324	0.000	0.031	0.700	0.355	0.000
H7: POS → EE	0.094	0.999	0.090	0.928	0.184	0.999
H8: POS → Self-efficacy	0.009	0.564	0.194	0.001	0.185	0.002

## Discussion and Conclusion

In this study, employees from Taiwan and Chinese mainland were adopted as research samples to examine the correlations among PK, POS, self-efficacy, SE, IB, and JP by means of the SCCT. The study contributes to filling the theoretical gap while applying Western theories in an Eastern context (Lent et al., 1994; Brown et al., 2011; Chang and Edwards, 2015), and increasing the generalization of the theory. Based on our results, this study is aimed at offering the following. First, there are few studies that have given a verification of employees’ IB based on an enormous environmental challenge (Thompson et al., 2016). In the study, the process of strengthening employees’ competence and IB in the context of the global pandemic have been investigated, and practical implications have been intended for corporate management. Second, despite most previous studies on SCCT accounting for the significance of environmental elements (Brown et al., 2011; Hansen et al., 2012; Duffy et al., 2013; Chang and Edwards, 2015; Liguori et al., 2019), merely a few studies showed essential contributions from worldwide environmental factors. This study intends to fill the theoretical gap and enrich the theoretical foundation of SCCT. Third, in addition to verifying the research framework established by SCCT in the Asian context, this study also provides intercultural perspective to compare differences among Taiwan, Chinese mainland, and Malaysia. There are more insights and advice regarding theories of human resources supplied by our results.

According to the results, the PK and POS of employees from Taiwan and Malaysia have a positive correlation with their self-efficacy and EE, but there is no significant effect on the paths of PK → self-efficacy and POS → EE on employees from Chinese mainland. These findings are in accord with those from Hansen et al. (2012), Lent et al. (2016), and Meyers et al. (2019); based on the SCCT, they consider that employees’ working state and attitudes can be affected by environmental deviations (Rehg et al., 2012), resulting in differences in the acquisition of capabilities and skills. Our results are consistent with those from previous studies to a substantial extent, which support the availability of the SCCT models in regions within a certain range (Hansen et al., 2012). Besides, as it is difficult for employees to be accessible to adequate psychological support provided by organizations (Schultz et al., 2015), supervisors or colleagues guided by the stagnation of business activities that develop proper EE and confidence for task fulfillment, inessential correlations may exist between the paths of PK → self-efficacy and POS → EE on employees from the Chinese mainland.

Furthermore, the positive correlations between the paths of PK → EE and POS → self-efficacy for employees from Taiwan, Malaysia, and the Chinese mainland have been revealed. It is also notable that according to the individual and organizational support mechanisms, employees possessing more PK and POS from organizations or supervisors are inclined to be more committed to the work surroundings and actively engaged in task activities, thus acquiring capabilities and confidence of task fulfillment, such as developing systematic/integrative minds and skills of resolving problems. The results are consistent with those from some previous studies (Schultz et al., 2015) which support the relationship existing between support mechanism and self-efficacy. Even though researchers have started with the examination of the connection among POS, work conditions, and work impetus in conformity with motivation theory (e.g., Schultz et al., 2015), and as far as we know, there are few previous studies which have investigated the influence brought to psychological and capability needs by individual or organizational factors. Therefore, the current research shows for the first time that the more the employees perceive a high level of the construction mechanism for IB (Gillet et al., 2012), the better they will meet their self-efficacy and EE.

In addition, it is shown that self-efficacy and SE make mass contributions to IB for employees from Taiwan, Malaysia, and the Chinese mainland. Moreover, self-efficacy plays a significant mediating function when it comes to the research model of SCCT. These results go in line with those from Lent et al. (2016) and Meyers et al. (2019) to a great extent, who conducted a cross-sectional verification of the IB model with diversified samples of employees (Hansen et al., 2012). Moreover, differing from the study made by Meyers et al. (2019), in this study, a comparison of samples from diversified regions with the same model, such as employees from Germany, Indonesia, Holland, Romania, and South Africa, is made, and a good comprehensive model-data fit in both samples is reported (Taiwan and Chinese mainland), and direct and indirect effects that self-efficacy generated in the IB model of SCCT bring to IB are verified. Nonetheless, different from the studies made by Lent et al. (2016) and Meyers et al. (2019), in this study, effects on the psychological aspect derived from worldwide environmental events are taken into consideration, and the theoretical model and SCCT of IB based on the regional analysis are enriched. Besides, the findings show that IB is found to have a positive and significant relationship with JP for employees from both Taiwan and the Chinese mainland. The result indicates that employees are significantly driven to enhance their JP in diversified work surroundings, particularly in a difficult situation, by positive psychological attitudes. The positive influence that IB brings to JP is consistent with the results from prior studies, which may enhance the utility of explanations and cultural associations of SCCT models to individuals living in various countries and cultures.

Through examining the extent to which regions where employees exist (Taiwan, Malaysia, as well as Chinese mainland) influence the correlations among POS, PK, self-efficacy, EE, IB, as well as JP, a theoretical contribution has been provided by the study. This goes in line with recent work done by Sheu and Bordon (2017), presenting that more attention has been offered to contextual support in international research on SCCT. It is found from the geographic distribution of international research on SCCT that Asian and European countries are still in need of more empirical attention. According to Sheu and Bordon (2017), cross-regional and cross-cultural differences are suggested to be included and explored in subsequent research. Through the test of a structural model across three groups, the structural relationships existing among the constructs are predicted to be stronger for transnational business administrators who have employees from Taiwan, Malaysia, and Chinese mainland. Nevertheless, the success of the PLS-SEM multi-group analysis indicates that the work surroundings are viewed as a moderator variable, showing that offline offices exist to enhance the relationships among PK, POS, self-efficacy, SE, IB, and JP.

### Practical Implications

Based on our results, this study suggests some significant practical implications to improve the quality of human resources. First, POS and PK were equally significant and predictive for employees’ own perceived degree of self-efficacy and EE, thus having an effect on IB. Building mechanisms of mentality that are individual and organizational are conducive to employees in terms of acquiring more resources and psychological support, which provide conditions essential for IB improvement. Thereby, as countries and regions worldwide are undergoing a struggle with the COVID-19 pandemic at present, when facing such similar events, organizations need to facilitate supervisors to establish a positively close connection with employees, set up platforms for communication via technological media and information technology devices, and offer real-time tasks or psychological support.

Second, external environmental elements, the worldwide pandemic of COVID-19 in particular, may affect employees’ work state. Thus, the examination for a sense of risk management is essential for managers. Based on this, companies or organizations are suggested to turn to preventive measures for risk management in this study to contend with threats and challenges arising from adaptive risks when encountering similar events. Even though all employees are prompted to engage in online working due to this event, not all employees possess technological media or information technology devices which are required. As a result, it is a necessity for managers to keep statistics on how many employees own information technology devices first and then figure out whether work tasks can be accomplished via online working; then the work tasks that fail to be accomplished via online work need to be rearranged in accordance with a schedule.

Third, in view of the structural patterns for three regions, IB originating from self-efficacy of employees from Taiwan and Malaysia is superior to that of employees from Chinese mainland. It is found that working online will influence employees. In regions that have been blocked for a longer time, employees are likely to feel more helplessness, disability, and anxiety. Even though employees are confident in task accomplishment, they are suffering from negative energy led by blockage. In this study, managers are suggested to provide support in other ways, such as opportunities, resources, and autonomy, to assist employees in conquering the threats and challenges from their surroundings and participate in their IB.

### Research Limitations

The research findings make contributions to the literature concerning employees in specific regions, SCCT, and employees’ IB. However, there are still some limitations existing and representing subsequent research directions. First, there is considerable status for SCCT in the field of psychology, but merely a few studies have taken the relationship between building mechanism and IB of employees into consideration. Despite this study referring to the SCCT and establishing the building mechanism, and significant organizational theories are available to be drawn from the findings, other motivation theories, including the theories of organizational learning, self-efficacy, and hierarchy needs, still apply to explaining how to stimulate IB for employees in a specific region. Therefore, subsequent research is suggested to apply diversified theoretical models to identifying related psychological dimensions that play an effect on employees’ IB. Second, employees are required to do a self-report of details regarding their mental building mechanism as the indicator in the study, which is largely attributed to the actual data that is confidential and not accessible in an easy way. Nonetheless, there may be errors occurring in employees’ self-statement of mental conditions. If the actual mental conditions of employees are assessed, the connection between building mechanism and IB may be better understood, considering research ethics. In addition, subsequent researchers are suggested to include contents of interviews and employees’ observations of work state into their studies to sustain the research findings and draw a comprehensive judgment. Third, restricted by time and space, a total of 1576 valid copies of the questionnaire were sampled. The research objects were classified into employees from Taiwan and Chinese mainland. Subsequent research can be made to both expand the quantity of samples and research representativeness, and conduct an exploration and comparison of other groups, so that extra insights related to organizational behavior management are offered.

## Data Availability Statement

The raw data supporting the conclusions of this article will be made available by the authors, without undue reservation.

## Ethics Statement

The studies involving human participants were reviewed and approved by Academic Committee of School of Economics and Management of Foshan University and University of Taipei. The patients/participants provided their written informed consent to participate in this study.

## Author Contributions

MP contributed to conception and design of the study. YX organized the database. CC performed the statistical analysis. MP wrote the first draft of the manuscript. All authors contributed to manuscript revision, read, and approved the submitted version.

## Conflict of Interest

The authors declare that the research was conducted in the absence of any commercial or financial relationships that could be construed as a potential conflict of interest.

## Publisher’s Note

All claims expressed in this article are solely those of the authors and do not necessarily represent those of their affiliated organizations, or those of the publisher, the editors and the reviewers. Any product that may be evaluated in this article, or claim that may be made by its manufacturer, is not guaranteed or endorsed by the publisher.
